# Early-onset of sexual activity as a potential risk of cervical cancer in Africa: A review of literature

**DOI:** 10.1371/journal.pgph.0000941

**Published:** 2023-03-22

**Authors:** Alemayehu Gonie Mekonnen, Yohannes Moges Mittiku

**Affiliations:** School of Nursing and Midwifery, Asrat Woldeyes Health Science Campus, Debre Berhan University, Debre Berhan, Amara Regional State, Ethiopia; University of Southern Denmark: Syddansk Universitet, DENMARK

## Abstract

**Introduction:**

In most African countries, cervical cancer is the most common cancer among women, both in terms of incidence and fatality. In the existing literature, age is risk factor for developing cervical cancer since it occurs mainly after the middle life of women. However, there have been contradictory findings in the literature on whether early sexual intercourse is linked to cervical cancer, with some studies indicating no relationship and others reporting an increased risk. Hence, this review analyzed data from recently published studies on cervical cancer.

**Methods:**

Seven databases (MEDLINE via PubMed, Google Scholar, Scopus, Medscape, EMBASE, African Journals Online and Science Direct) were searched for papers published from January 2000 to March 2022 in English. Ten studies were included in analysis. The statistical analysis was performed using STATA 11. Heterogeneity between-study was explored by forest plot and inconsistency index (I^2^). The publication bias was checked by a funnel plot and Egger’s test. The pooled estimates of odds ratios were calculated by a random-effects model.

**Results:**

In the subgroup analysis, there was no significant association between early sexual activity and cervical cancer. However, the overall pooled analysis of these ten studies revealed that there is an association between early sexual debut and cervical cancer. In the random effect model, we found a pooled odds ratio of 2.95 (95% CI = 1.06, 4.83), indicating that women who began sexual intercourse before the age of 18 had a higher risk of getting cervical cancer than adult women.

**Conclusion:**

In this analysis, women who began having early sexual debut had a greater risk of developing cervical cancer than those who initiated sexual intercourse later in life. Delaying the age of first sexual activity among adolescents could help prevent the occurrence of cervical cancer.

## Introduction

Cervical cancer is the second most common cancer in women, and it is a major public health concern worldwide, accounting for 16% of all female cancers [1–5]. Every year, a high number of new cases of cervical cancer are diagnosed, and more than half a million cervical cancer deaths reported annually [6,7]. Alarmingly, developing countries accounted for the majority of new cervical cases [8,9]. In most African countries, cervical cancer is the most common cancer among women, both in terms of incidence and fatality [7,10].

Several risk factors for cervical cancer have been identified as of yet. Cigarette smoking has long been recognized as the main risk factor for cervical cancer through genotoxic and immunosuppressive mechanisms [11,12]. Infection with the human papillomavirus (HPV) has also been confirmed as a proven risk factor for the development of cervical carcinoma in Africa [12–15], where HPV infection is more endemic and problematic than in high-income countries [13–19]. Indeed, having sexually transmitted diseases [20,21], early marriage [11,22], early sexual debut [23,24], all of which are the index of developing countries including Africa, were found to be possible risk factors for cervical cancer.

Interestingly, the available literature consistently showed age is another risk factor for developing cervical cancer since cervical cancer occurs mainly after the middle life of women [15,25,26]. However, there has been some conflicting findings in the literature about whether early sexual intercourse is linked to cervical cancer [27]. A number of individual studies reported varying results, with some indicating no link between an early sexual debut and an increased risk of cervical cancer, and others indicating a link between an early sexual debut and an increased risk of cervical cancer. For example, no association was identified between early age at first intercourse and cancer risk in studies that adjusted for the number of sexual partners [28,29]. Similarly, studies conducted in Finland [30], Mali [31], and Ethiopia [32] found no relationship between early initiation of sexual activity at a young age and the risk of cervical cancer. On the other hand, the diagnosis of cervical cancer at young age was associated with an early sexual debut [33].

As such, these individual studies have limited statistical power and are relatively unreliable because they have small sample sizes or scope limitations, have been reported using similar procedures, and, in some cases, lack adjustment for confounding factors. Therefore, the association between early sexual activity and development of cervical cancer is yet unclear. To provide strong evidence of whether the early onset of sexual activity is a risk of cervical cancer, we decided to analyze data from recently published literature of risk factors for cervical cancer. This systematic review can add to the existing body of knowledge by providing a comprehensive estimate of early sexual activity as a risk factor for cervical cancer. The pooled estimations could contribute to identify sexually active adolescents for the purpose of delaying the age of first intercourse and initiating routine Pap tests so that early prevention can be effective.

## Methods

### Search strategy

The overarching research hypothesis was; “early-onset of sexual activity is a risk for developing cervical cancer. The chance of developing cervical cancer is higher among women who had started sex before the age of 18 years”.

**Fig 1:**
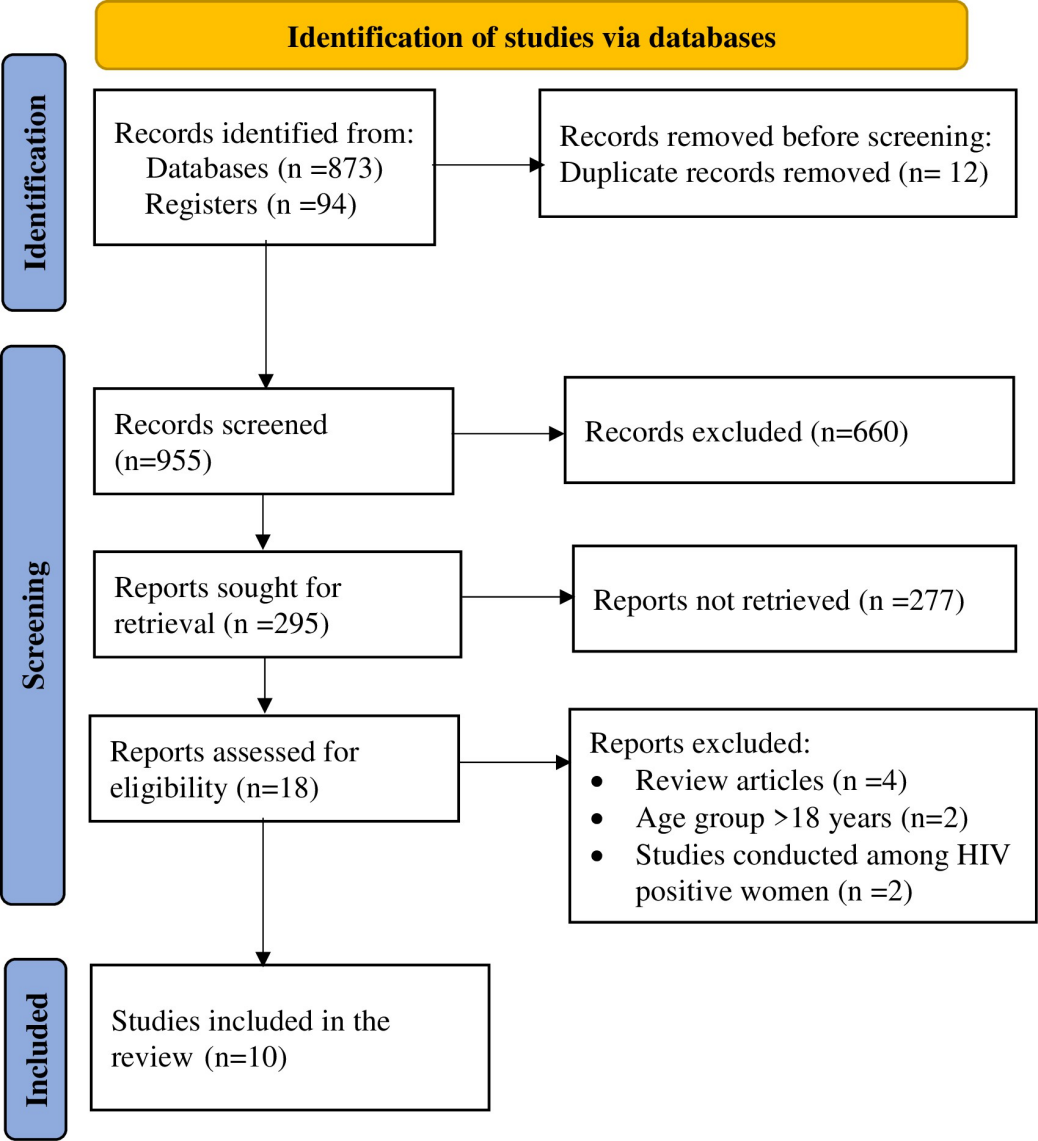
PRISMA flow-diagram that depicts the phases of study selection, March 2022.

Preferred Reporting Items for Systematic Reviews and Meta-Analyses (PRISMA 2020) flow diagram [34] was followed to report the findings of this review (Fig 1). This review covered all publications investigating the risk of cervical cancer. Seven databases (MEDLINE via PubMed, Google Scholar, Scopus, Medscape, EMBASE, African Journals Online and Science Direct) were searched for papers published from January 2000 to March 2022 in English. A broad search was deliberately conducted to ensure all papers would be retrieved. The search was supplemented by hand searching. In addition, we reviewed the citations and reference lists of identified studies to identify articles that were not captured by the database searches. To identify relevant articles, titles and abstracts of retrieved articles were exported to Endnote to screen duplicate articles. Then the review authors assessed and reviewed independently all studies deemed suitable to determine inclusion.

The following search terms were employed to search for articles from major databases mentioned above: (early OR adolescent OR less than 18 years old) AND (sexual activity OR sexual debut OR sexual initiation OR sexual intercourse OR sexual behavior OR sexuality) AND (risk factors OR factors OR cause OR etiology OR causality OR determinants) AND (cervical cancer OR invasive neoplasia OR cervical lesion OR cervical adenoma OR cervical dysplasia OR cancer of the cervix OR Intraepithelial lesion) AND (case-control OR Cross-sectional OR cohort studies). These key terms were combined using Boolean operators “AND” and “OR” to narrow the search. We also limited our search to peer-review literature as it guarantees quality checks.

### Inclusion and exclusion criteria

To meet the inclusion criteria, titles and abstracts of studies were examined, and the following study selection criteria were applied for considering studies for this review.

#### Types of studies

Full-text case-control, cross-sectional cohort studies were included.

#### Participants

This study included studies conducted among African women populations. This study was restricted to African countries because the sexual behavior of this population was varied from developed regions and other parts of the world (for instance developed countries have late age at first sexual intercourse [35,36] and the study site was therefore excluded from this analysis. Besides, early marriage is a culture and commonly practiced in the African population which has a relation with the development of cervical cancer [15].

#### Time frame

This review included studies that were published after the year 2000. Because, besides minimizing the time-lag bias, cervical cancer is becoming the common reproductive health problems in Africa in recent years.

#### Definitions

In this systematic review and meta-analysis, early onset of sexual activity/intercourse was defined as having the first sexual intercourse at the age of 18 years or younger [37].

#### Exclusion criteria

In the current meta-analysis, review studies, studies which did not include adjusted odds ratios (AORs) and their 95% confidence intervals (CI) were excluded. Studies involving women over the age of 18 were excluded because early sexual activity is defined as having sexual intercourse at the age of 18 or younger. Moreover, studies published other than the English language were excluded from the study.

### Data extraction and management

The authors screened all identified titles/abstracts. Articles found relevant by title and abstract were undergone for full-text review for eligibility. For eligible studies, two authors extracted the data using the pre-determined inclusion criteria. All steps of the data extraction process were carried out blindly. The review authors recorded the data on Microsoft excel and extracted the following data for each study: the authors, year of publication, study setting, study design, sample size, and quality of publication (Table 1). The study selection and data extraction were done from January 2000 to March 2022. Two review authors (AGM and YMM) worked independently to extract data using a pre-specified data extraction form. Discrepancies in data extraction were resolved through discussion, and articles were included once consensus was reached.

**Table 1:** Summary of the included studies, March 2022.

Authors	Design	Sample size				Country	Regions	Quality of the article
		Diagnosed with cervix cancer		Have no cervical cancer				
		Had sexual debut	Total cases	Had sexual debut	Total control			
Makuza JD et al [9]	Cross-sectional	37	406	28	445	Rwanda	East Africa	6 star
El-Moselhy EA et al [19]	Case-control	28	86	38	200	Egypt	East Africa	7 star
Cooper D et al [42]	Case-control	168	524	357	1541	South African	South African	6 star
Hammouda D et al [43]	Case-control	63	136	51	200	Algeria	North Africa	6 star
Kassa RT [44]	Case-control	25	55	20	109	Ethiopia	East Africa	6 star
Ogunbowale T et al [45]	Cross-sectional	42	138	26	140	Nigeria	West Africa	6 star
Bezabih M et al [46]	Case-control	6	60	28	120	Ethiopia	East Africa	7 star
Bayo S et al [47]	Case-control	63	82	72	97	Mali	West Africa	8 star
Hailemariam T et al [48]	Cross-sectional	270	1220	51	725	Ethiopia	East Africa	7 star
Utoo BT et al [49]	Cross-sectional	10	139	6	69	Nigeria	West Africa	7 star
**Total**		**712**	**2846**	**677**	**3646**			

### Quality appraisal of the primary studies

We used the Newcastle-Ottawa Scale (one of the most widely used guideline for reporting observational studies worldwide) [38,39] for quality assessment of the selected studies [40]. Each element of quality assessment was labelled as: 1 = a criteria was met and 0 = a criteria was not met. A study was considered a very good study when the sum of met criteria is 9–10, a good study when the sum of met criteria is 7–8, and satisfactory when the sum of met criteria is 5–6. All the included studies scored above 6 and are included in the analysis.

### Data analysis

The statistical analysis was performed using STATA 11 (meta-analyses package; Stata Corporation, College Station, TX, USA). Heterogeneity between-study was explored by forest plot (a visual technique that checks whether the confidence intervals of studies overlap with each other) and inconsistency index (I^2^) (a statistical method which describes the percentage of total variation across studies). The I^2^ provides the percentage of variability due to heterogeneity rather than the chance difference or sampling error. The I^2^ greater than 75% and Chi^2^ test (P<0.10) was considered statistically significant heterogeneity. Pooled estimates of odds ratios were calculated by a random-effects model with a 95% confidence interval. The random-effects model which assesses the variability within and between studies was applied to estimate odds of developing cervical cancer among females who had started the early sexual activity. Besides, random effect minimizes heterogeneity of the various included studies than the fixed effect model [34].

The publication bias was assessed using the funnel plot (which displays effect sizes plotted against the sample size, standard error, conditional variance, or some other measure of the precision of the estimate) and Egger’s test. In the presence of a cloud of data points that is symmetric around the population effect size and has the shape of a funnel, one can conclude as no publication bias [41]. Statistical significance of publication bias was declared if the p-value for Egger’s test was <0.05. Sensitivity analysis, using a random-effects model, was performed to assess the influence of a single study on the overall meta-analysis or the overall estimates. Besides, to be free of other sources of bias (selection bias, performance bias, and reporting bias), review authors independently assessed risk of bias in included studies and resolved discrepancies through discussion.

## Results

### Characteristics of included studies

In this search for published studies, 967 records were identified through the database search and cross-referencing. From these studies, 481 articles were removed using their title review and 179 were removed using abstract review. Following title and abstract review, 307 studies were potentially eligible for full-text review, and 289 research articles were excluded after the full-text review, with the majority not meeting the inclusion criteria. Finally, 10 studies [9,19,42–49] comprising of 6492 participants were included in this pooled analysis (Fig 1).

Of the included articles, 7 studies were case-control studies [9,19,42–44,46,47] and 3 studies were cross-sectional [45,48,49]. The included studies were published from 2002 [47] to 2018 [44] and six (60.0%) of them were published after 2010 [9,19,44,46,48,49]. The total numbers of cervical cancer cases were 2846 while 3646 women were free from cervical cancer. Out of the total participants, 1389 women (21.4%) had been engaged in early sexual intercourse. All the studies included African populations, with five studies were conducted in East Africa [9,19,44,46,48], three were in West Africa [45,47,49] and the rest two studies were from South African [42] and North Africa [43]. In this meta-analysis, the quality of the studies was evaluated by using the Newcastle-Ottawa quality scale [38]. All studies achieved a score of at least six stars, indicating good study quality of included studies. Relevant features of each study; the author, study design, sample size, country, regions and the quality of the article were summarized in Table 1.

### Heterogeneity

The included studies were assessed for heterogeneity. Accordingly, the studies were significantly heterogeneous and the true variability among the 10 studies other than chance was 98.9% (I^2^ = 98.9%, p = 000). Besides, the visual inspection of the funnel plots suggested that the potential sources of the heterogeneity could be attributed to the study by Cooper D et al [42]. This considerable heterogeneity may be due to methodological quality.

### Publication bias

**Fig 2:**
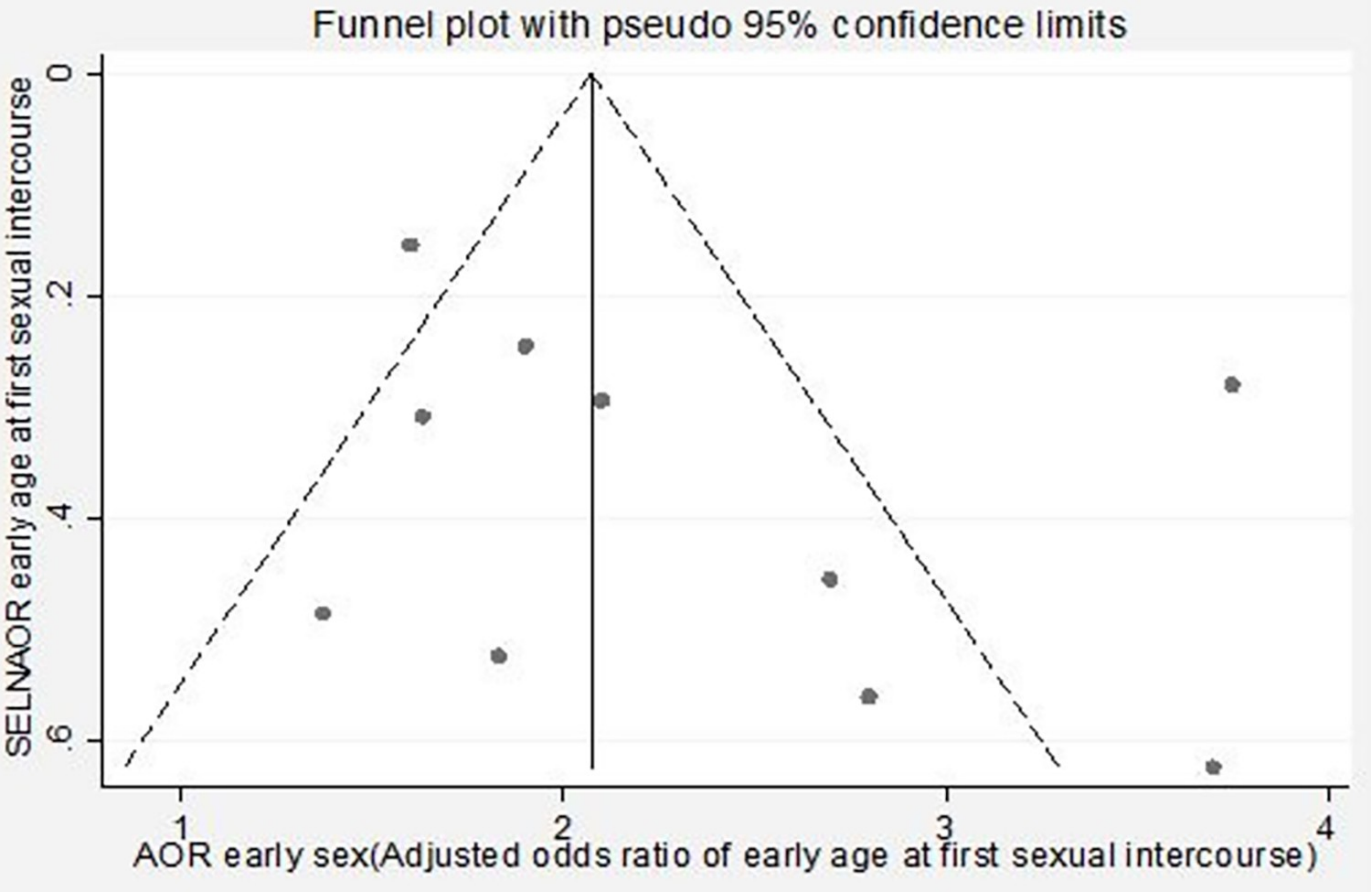
Funnel plot showing publication bias (inverted symmetrical funnel plot), March 2022.

The publication bias was checked by using the visual inspection of the funnel plots and the Egger’s test, and the plot has a symmetric inverted funnel shape showing no evidence of variability in effect sizes from studies and publication bias (Fig 2). Egger’s test also provides no evidence for small-study effects and publication bias among studies (giving a p-value of 0.926).

### Sensitivity analysis

**Fig 3:**
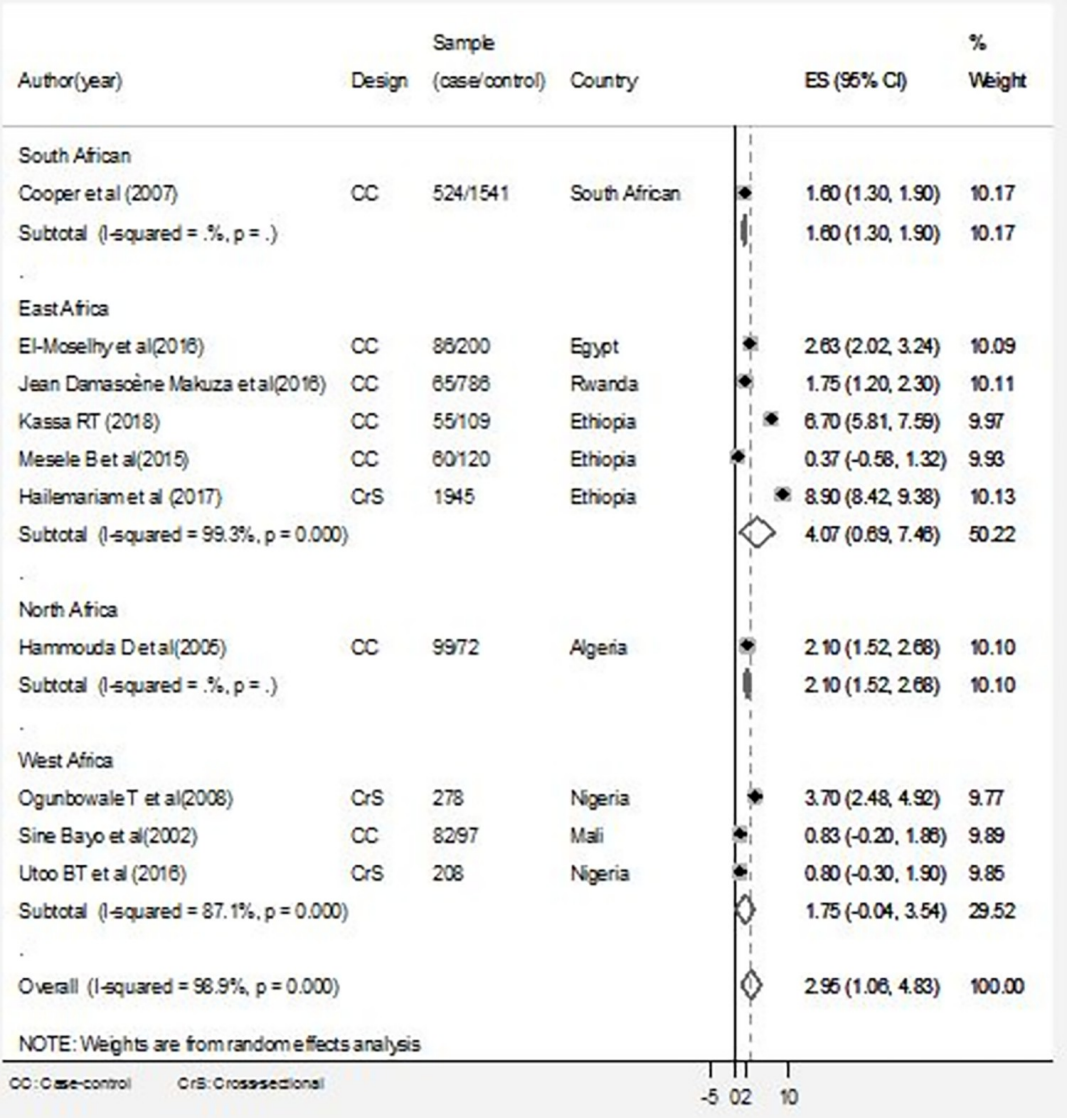
Forest plot of pooled estimates of the association between early sexual intercourse and having cervical cancer: Subgroup analyses by regions, March 2022.

A sensitivity analysis was also conducted to test whether a particular study was responsible for the presence of high heterogeneity. The output showed that the estimated points of the sensitivity analysis were within the confidence interval for the pooled estimate of the meta-analysis that shows no statistical source of heterogeneity among the studies (Fig 3).

### Subgroup analyses

To resolve the presence of heterogeneity, subgroup analysis using the random-effect model was employed. In this analysis, 10 studies were assessed to see if there was an association between early sexual activity and the risks of cervical cancer. The adjusted odds ratios of the articles included in the study ranged from 0.37 (95% CI = -0.58, 1.32) [46] to 6.70 (95% CI = 5.81, 7.59) [44]. In the subgroup analysis, by using regions as a stratification of studies, high heterogeneity was detected among East African studies (I^2^ = 99.3%, P = 000). As shown in Fig 3, there was no significant association between early sexual activity and cervical cancer in these five east African countries (random effect OR = 4.07, 95% CI = 0.69–7.48). Similarly, there was considerable heterogeneity among West African studies (I^2^ = 87.1%, P = 000), and early sexual activity was not associated with cervical cancer (random effect OR = 1.75, 95% CI = -0.04–3.54). However, the overall pooled analysis of these ten studies showed that there is an association between early sexual intercourse and having cervical cancer. We found a pooled odds ratio of 2.95 (95% CI = 1.06, 4.83) in the random effect model, indicating that women who began sexual intercourse before the age of 18 had a greater risk of getting cervical cancer (Fig 3). This result, however, should be interpreted cautiously because the study design, methods of analysis varied from study to study, and there was evidence of statistical heterogeneity between studies that influences the pooled estimates of the odds ratio.

## Discussion

This systematic review and meta-analysis, to the best of our knowledge, is the first comprehensive meta-analysis conducted in the African population to determine the association between the early sexual activity and its risk of cervical cancer. Our findings contribute to the growing body of evidence by indicating that early sexual engagement is associated with an increased risk of cervical cancer. Additionally, the findings can be used to support intervention strategies that focus on delaying the age of first sexual intercourse and instituting routine Pap testing in order to achieve effective cervical cancer prevention.

Cervical cancer is becoming more common in resource-limited countries, which also account for the majority of cervical cancer deaths [9,10]. This is perhaps unsurprising given the high rate of early marriage reported in developing countries, particularly in Africa [50,51], and the fact that early marriage is linked to early sexual activity. In fact, substantial studies also suggested that early marriage, which is linked with early sexual activity, has been associated to the development of cervical cancer [51,52].

In this systematic review and meta-analysis, women who began sexual intercourse before the age of 18 years had a greater chance of having cervical cancer than women who initiated sexual intercourse after the age of 18 years. The pooled estimate showed that women who had sexual intercourse before the age of 18 years had 2.95 times higher risk of acquiring cervical cancer than women who began sexual intercourse later in life. This finding is in agreement with a study conducted among Chinese women [53]. The association between early sexual debut and the incidence of cervical cancer was also revealed in the re-analysis of individual data from 21 epidemiological studies [54] and other several individual studies [14,55,56]. This connotation is not surprising because the increased risk of cervical cancer among adolescents who initiated early sexual debut might be due to the immature cervix that is suitable for HPV infection; a confirmed carcinogenic agent to the cervical epithelium [57,58]. Indeed, sexual activity before the age of 18 has been linked to an increased risk of HPV infection, which can lead to cervical dysplasia and abnormalities [37].

Another possibility is that HPV infection among adolescents is high because their sexual partners are more likely to be adult men who have had multiple sexual partners and have been exposed to HPV infections [37]. As evidenced by available studies, adolescents who had sexual intercourse with HPV-positive men are more likely to become infected with the virus, which again exposed them to cervical cancer [27,37].

In subgroup analyses stratified by regions, however, there was no association between early sexual intercourse and the risks of having cervical cancer. Remarkably, we found no evidence of significant association between early sexual activity and cervical cancer in studies conducted in East and West African countries. Even though individual studies lacked statistical power and were relatively heterogeneous, majority of them reported the relationship between early sexual intercourse and having cervical cancer. In contrast to our findings, for example, a study published in 2009 reported evidence of a relationship between cervical cancer and sexual intercourse before the age of 16 years [52]. The relationship of early sexual intercourse as a risks of cervical cancer was also reported previous studies [50,59,60]. This difference could be explained by the regional and methodological variance of studies in the risk of cervical cancer. Furthermore, the variations of the findings are likely arisen from the inherent limitations of the literature, and the result should again be interpreted with caution due to the small sample sizes of included studies and evidence of statistical heterogeneity between studies, which can influence the pooled estimates.

## Limitations of the study

Even though we pooled the adjusted estimates of individual studies and retrieved studies that adjusted for HPV negative results to minimize confounding by HPV infection, this meta-analysis may have some other limitations. We were unable to determine whether the included studies had been adjusted for the independent risk of cervical cancer since the specific adjustment factors vary from study to study. Since the included studies were restricted in reports published in English, important data sources might be missed. The authors also acknowledge the methodological limitation of the included studies and unknown biases.

## Conclusion

In this analysis, women who began having early sexual debut had a greater risk of developing cervical cancer than those who initiated sexual intercourse later in life. Delaying the age of first sexual activity among adolescents could help prevent the occurrence of cervical cancer.
